# Spirulina-Derived Carbon Dots Promote Context-Dependent Effects on Rice Metabolism, Yield, and Grain Quality Under Non-Stress and Heat Stress Conditions

**DOI:** 10.3390/plants15111657

**Published:** 2026-05-28

**Authors:** Luana Vanessa Peretti Minello, Shaiane Lessa dos Santos, Luana Bueno Longaray, Natan da Silva Fagundes, Sidnei Deuner, Aline Nunes, Eva Regina Oliveira, Marcelo Maraschin, Raul Antonio Sperotto

**Affiliations:** 1 Graduate Program in Plant Physiology, Federal University of Pelotas, Pelotas 96010-610, Brazil; lvpminello@gmail.com (L.V.P.M.); buenolongaray@gmail.com (L.B.L.); natanfagundes@gmail.com (N.d.S.F.); sdeuner@yahoo.com.br (S.D.); 2 Botany Department, Biology Institute, Federal University of Pelotas, Pelotas 96010-610, Brazil; shaianelessadossantos44@gmail.com; 3 Institute of Biosciences, São Paulo State University, Botucatu 18618-689, Brazil; alinenunes_bio@hotmail.com; 4 Laboratory of Metabolomics and Applied Biochemistry, Federal University of Santa Catarina, Florianopolis 88040-900, Brazil; ginagro@gmail.com (E.R.O.); mtocsy@gmail.com (M.M.); 5 Brazilian Agricultural Research Corporation (Embrapa Maize and Sorghum), Sete Lagoas 35702-098, Brazil

**Keywords:** climate changes, nanotechnology, carbon dots, heat stress, field experiment, grain yield

## Abstract

Carbon dots (CDs) have emerged as promising nanobioinputs capable of modulating plant metabolism and stress responses. However, their effectiveness under field conditions remains poorly understood. This study evaluated the effects of Spirulina-derived carbon dots (S-CDs) on rice metabolic, agronomic, and grain-quality responses under non-stress and heat stress conditions. Two independent field experiments were conducted under ambient and heat stress conditions, the latter imposed using temporary greenhouse structures during the reproductive stage. S-CDs were applied by foliar spraying (0.2 mg mL^−1^) at key developmental stages. Their effects were assessed through metabolic, agronomic, and grain quality analyses. Under non-stress conditions, daily average temperatures ranged from 20 to 27 °C, while daily maxima ranged from 24 to 38 °C. Heat stress increased daily average temperature by 3.9 °C and daily maximum temperature by 12.5 °C, with temperature peaks frequently exceeding 45 °C for several hours. Under non-stress conditions, S-CDs induced modest changes in antioxidant, carbon, and nitrogen metabolism, but without consistent improvements in grain yield or yield components. Under heat stress, however, S-CD application reduced spikelet sterility and increased grain yield despite limited changes in the metabolic variables evaluated. Grain quality and nutritional composition also responded differently depending on the environmental condition, indicating context-dependent effects. S-CDs showed limited agronomic relevance under favorable field conditions, but contributed to yield stability under heat stress. These findings support the potential of S-CDs as complementary nanobiostimulants to improve rice resilience under climate-related stress conditions.

## 1. Introduction

The growing demand for food and increasing climate variability challenge agriculture and highlight the need for innovative technologies capable of sustainably enhancing crop productivity, grain quality, and stress resilience under adverse environmental conditions [1]. Among these technologies, carbon-based nanoparticles like carbon dots (CDs) have attracted attention due to their potential to improve plant performance and productivity [2]. Previous studies show that CDs can interact with plant systems and affect plant growth, stress tolerance, and grain quality [3,4,5]. However, substantial knowledge gaps remain, particularly regarding their effects on staple crops such as rice under realistic field conditions.

Among staple crops, rice (*Oryza sativa* L.) is one of the most important cereal species worldwide. It has high economic and nutritional value and plays a central role in global food security [6]. Cultivated in more than 100 countries, rice provides approximately 20% of global calorie intake [7,8]. Flooded rice systems usually ensure stable yields due to adequate water availability and management practices. However, increasing climate variability, particularly fluctuations in temperature and rainfall, can substantially reduce productivity, especially during the reproductive stage [9,10]. High temperatures during anthesis (>35 °C, even for short periods) disrupt critical reproductive processes such as anther dehiscence, pollen viability and germination, and pollen tube growth. As a result, spikelet fertility declines and severe stress may even cause crop failure [11,12]. Under these conditions, spikelet fertility can decline by 30–80%, and yield losses may reach approximately 6% per °C per day of stress [13,14]. Heat stress also affects key physiological and metabolic processes, including photosynthesis, carbon allocation, and redox balance. These changes ultimately impair grain filling and quality by altering carbohydrate partitioning and reducing starch accumulation [15].

These constraints demonstrate that rice reproductive development is highly vulnerable to heat stress and highlight the need for strategies capable of enhancing crop resilience under field conditions. In this context, nanostructured biostimulants have emerged as promising tools to improve plant performance and tolerance to adverse environmental conditions [2,16]. Among them, CDs have gained increasing attention because they can modulate plant responses to abiotic stresses, including heat stress [17]. Their small size, high surface reactivity, and biocompatibility make them attractive candidates for agricultural applications [18]. Controlled studies show that CDs can influence physiological and metabolic pathways linked to stress acclimation [17,18,19,20]. However, whether these responses are consistently maintained under field conditions remains unclear because environmental variability and source-sink dynamics strongly influence plant performance.

This study evaluated the field effects of CDs on the metabolic and agronomic performance of rice under non-stress and heat stress conditions. We applied Spirulina-derived carbon dots (S-CDs) via foliar spraying at key developmental stages and assessed their effects on plant metabolism, yield components, and grain quality. We imposed heat stress during the reproductive phase (R_4_) to test whether S-CD application modulates plant responses to high temperature. We hypothesized that S-CDs act as context-dependent modulators of plant metabolic responses under both non-stress and heat stress conditions, attenuating stress-induced disruptions during the reproductive stage and contributing to the maintenance of reproductive performance and yield stability in rice. By combining biochemical and agronomic analyses, this study provides a field-based assessment of how S-CDs affect rice performance under contrasting environmental conditions.

## 2. Materials and Methods

### 2.1. Synthesis and Characterization of Spirulina-Derived Carbon Dots (S-CDs)

S-CDs were synthesized as previously described by [21], with minor modifications. Briefly, the Spirulina biomass was carbonized at 300 °C for 2 h, and the resulting material was processed to obtain water-dispersible carbon nanoparticles. Their physicochemical properties, including morphology, elemental mapping, surface functional groups, particle size distribution, and zeta potential, were previously characterized by [22].

### 2.2. Experimental Site and Plant Material

**Figure 1:**
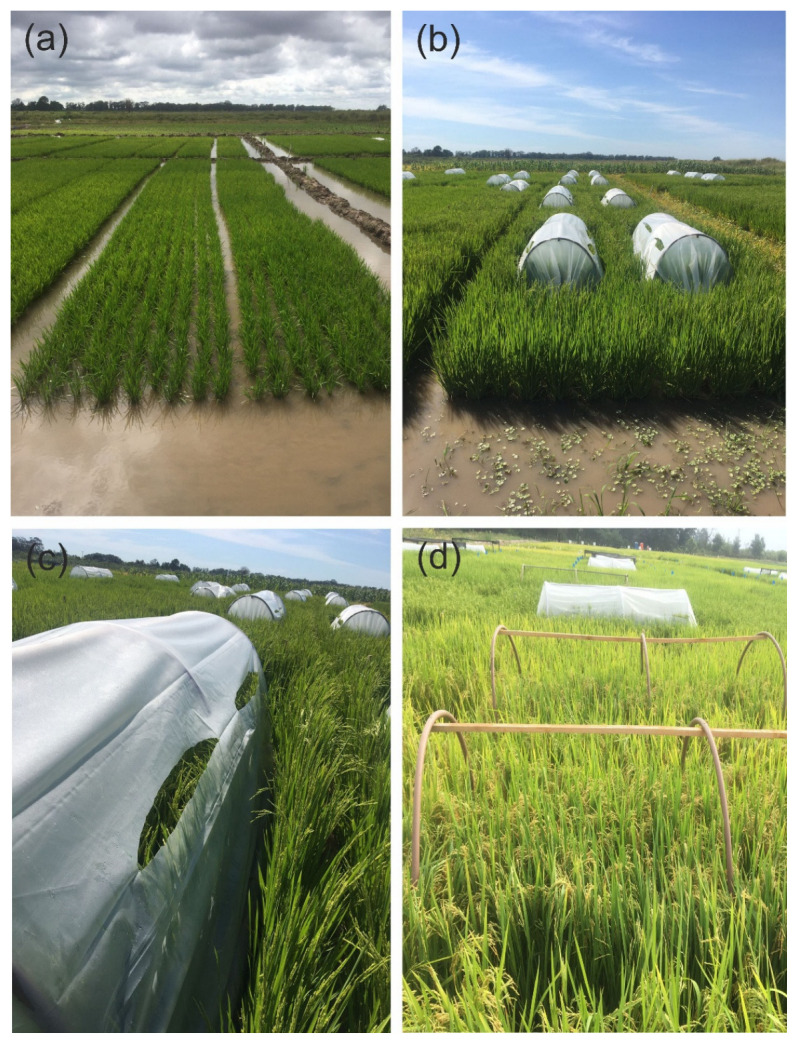
Experimental area at the Federal University of Pelotas during the first experiment under non-stress conditions (**a**) and second experiment under heat stress conditions (**b**). In the second experiment, temporary greenhouse structures (1.3 m × 3.0 m × 5.0 m; H × W × L) were used to impose heat stress during the early reproductive stage of rice for 16 days (**c**). Panel (**d**) shows the same structures after removal of the plastic cover.

Field experiments were conducted at the Palma Agricultural Center, Federal University of Pelotas, in Capão do Leão, Rio Grande do Sul, Brazil (31°48′07.9″ S, 52°30′17.9″ W; ~14 m a.s.l.). Two field experiments were established. The first was conducted under non-stress conditions (Figure 1a) and the second under heat stress conditions (Figure 1b). The soil in the experimental area is classified as a Haplic Planosol with carbonate characteristics, which is typical of coastal lowland regions [23]. Before the experiments were established, soil chemical properties at the 0–20 cm depth were determined according to [24] and are shown in Supplementary Table S1. The rice cultivar IRGA 424 RI was selected due to its high adaptability to regional climate. Seeds were sown at a density of 300 plants m^−2^ (120 kg ha^−1^) with 0.35 m between rows. Crop management followed the technical recommendations of [25].

### 2.3. Experimental Design, Treatments, and Sampling Strategy

#### 2.3.1. Field Application of CDs Under Non-Stress Conditions

**Figure 2:**
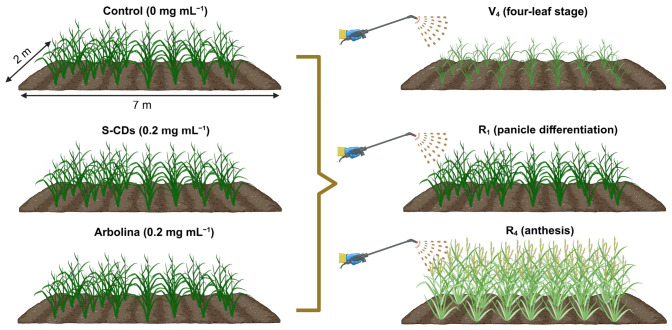
Experimental design of the field trials conducted with rice (*Oryza sativa* L. cv. IRGA 424 RI). Plants were foliar-sprayed with Spirulina-derived carbon dots (S-CDs) or Arbolina (commercial product) at 0.2 mg mL^−1^ at the V_4_, R_1_, and R_4_ stages, alongside an untreated control. Heat stress was imposed at the R_4_ stage using temporary greenhouse structures (1.3 m × 3.0 m × 5.0 m; H × W × L) for 16 days. In the non-stress experiment, biochemical measurements were performed 24 h after the third application. In the heat stress experiment, biochemical measurements were performed 7 days after stress imposition. The experiments followed a randomized block design with four independent replicates.

The experiment followed a randomized complete block design with four replicates. Experimental plots measured 2 m × 7 m (14 m^2^) and were separated by levees to prevent water movement between plots. Treatments consisted of T1: control (untreated); T2: Spirulina-derived carbon dots (S-CDs); and T3: Arbolina (commercial CD-based product) (Figure 2), with four replicates per treatment. CDs were applied via foliar spraying at a concentration of 0.2 mg mL^−1^ using a backpack sprayer calibrated to deliver 200 L ha^−1^ at three developmental stages: V_4_ (four-leaf stage), R_1_ (panicle differentiation), and R_4_ (anthesis) (Figure 2).

#### 2.3.2. Field Application of CDs Under Heat Stress (HS) Conditions

This experiment used the same plot dimensions and agronomic management described before. Treatments consisted of T1: heat stress (HS); T2: Spirulina-derived carbon dots (S-CDs) under HS; and T3: Arbolina under HS, with four replicates per treatment. HS was imposed at the R_4_ stage (anthesis) by covering the plots with temporary greenhouse structures (1.30 m height × 3.0 m width × 5.0 m length) (Figure 1c). The structures remained for 16 days to increase air temperature above ambient conditions. Air temperature inside and outside the structures was monitored using AK170 dataloggers (Akso, São Leopoldo, Brazil). After the stress period, the structures were removed (Figure 1d), and plants remained under field conditions until harvest.

### 2.4. Leaf Sampling and Sample Preparation

In the non-stress experiment, leaves were sampled 24 h after the third foliar application of CDs, at the R_4_ (anthesis) stage. In the HS experiment, leaves were sampled after 7 d of HS exposure. In both experiments, each sample consisted of five fully expanded leaves collected from five plants within each replicate. Leaves were taken from the position immediately below the flag leaf, pooled, frozen in liquid nitrogen, ground to a fine powder, and stored at −80 °C until analysis. Given the distinct objectives and sampling times of each experiment, the experiments were interpreted independently, aiming to assess the effects of CDs under non-stress and HS conditions, respectively.

### 2.5. Determination of Antioxidant Compounds and Antioxidative Capacity

#### 2.5.1. Total Phenolic Content (TPhnlcC)

TPhnlcC was determined as described by [26], with minor modifications. Phenolic compounds were extracted from 300 mg of powdered leaves using 3 mL of 80% methanol. For the reaction, 100 μL of the extract was mixed with 75 μL of Folin–Ciocalteu reagent and 825 μL of 2% sodium carbonate. After 1 h of incubation, absorbance was measured in triplicate at 750 nm using a microplate reader (ThermoPlate, model P-reader; Dallas, TX, USA). Gallic acid was used as the standard at concentrations ranging from 7.81 to 500 µg mL^−1^ and TPhnlcC was calculated from the calibration curve (y = 0.006x, r^2^ = 0.9967).

#### 2.5.2. Total Flavonoid Content (TFC)

TFC was quantified as described by [27], with modifications. Flavonoids were extracted from 300 mg of powdered leaves with 3 mL of 80% methanol. For the assay, 500 μL of the extract was mixed with 2.5 mL of analytical-grade ethanol and 500 μL of 2% aluminum chloride solution in methanol. After 1 h of incubation, absorbance was measured in triplicate at 420 nm using a microplate reader (ThermoPlate, model P-reader; Dallas, TX, USA). Quercetin was used as the standard at concentrations ranging from 7.81 to 500 µg mL^−1^ and TFC was calculated from the calibration curve (y = 0.0056x, r^2^ = 0.9799).

#### 2.5.3. Total Carotenoid Content (TCrtnC)

TCrtnC was determined as described by [28], with modifications. Carotenoids were extracted from 300 mg of powdered leaves with 3 mL of 80% methanol. Then, 300 μL of the extract was transferred to the microplates, and absorbance was measured in triplicate at 450 nm using a microplate reader (ThermoPlate, model P-reader; Dallas, TX, USA).

#### 2.5.4. Antioxidant Capacity

Antioxidant capacity was evaluated using the DPPH (2,2-diphenyl-1-picrylhydrazyl) radical scavenging assay, as described by [29]. Antioxidant compounds were extracted from 300 mg of powdered leaves with 3 mL of 80% methanol. The DPPH stock solution was prepared by dissolving 7.9 mg of DPPH in 2.5 mL of methanol. The working solution was prepared by diluting 0.5 mL of stock solution in 49.5 mL of 80% methanol and adjusting the absorbance to 0.5–0.6. For the reaction, 10 μL of the extract was added to 290 μL of the working solution. After 30 min of incubation, absorbance was measured at 540 nm using a microplate reader (ThermoPlate, model P-reader; Dallas, TX, USA). DPPH radical scavenging capacity was calculated as:

$$\left(\mathrm{Antioxidant}\;\mathrm{capacity}\;(\%)\right)\;=\left(\frac{\left(Control\;absorbance\text{-}Sample\;absorbance\right)}{Control\;absorbance}\right)\times \;100\%$$

where Control absorbance is the absorbance of the diluted DPPH, and Sample absorbance is the absorbance of the reaction mixture containing DPPH and extract.

### 2.6. Determination of Primary Metabolites

#### 2.6.1. Total Soluble Sugars (TSS) and Total Starch (TS)

TSS and TS were quantified according to [30]. For TSS determination, 50 mg of powdered leaves was extracted with 2 mL of MCW solution (methanol:chloroform:distilled water, 12:5:3, *v*/*v*/*v*). After centrifugation, 1 mL of chloroform and 1.5 mL of distilled water were added to induce phase separation. Then, 1 mL of the supernatant was collected, mixed with 2 mL of 0.2% anthrone in sulfuric acid, vortexed, and heated in a water bath at 100 °C for 3 min. Samples were cooled to room temperature, and absorbance was measured at 630 nm using a microplate reader (ThermoPlate, model P-reader; Dallas, TX, USA). Glucose was used as the standard at concentrations ranging from 62.50 to 2000 μg mL^−1^ and TSS was calculated from the calibration curve (y = 0.0018x, r^2^ = 0.9517).

For TS determination, the pellet remaining after TSS extraction was resuspended in 2 mL of 30% perchloric acid and centrifuged at 4000 rpm for 10 min. The supernatant was collected, and this step was repeated once. The final volume was adjusted to 4 mL with perchloric acid. Then, 1 mL of the extract was mixed with 2 mL of 0.2% anthrone in sulfuric acid, heated in a water bath at 100 °C for 3 min, and cooled to room temperature. Absorbance was measured at 630 nm using a microplate reader (ThermoPlate, model P-reader; Dallas, TX, USA). Glucose was used as the standard at concentrations ranging from 62.50 to 2000 μg mL^−1^ and TS was calculated from the calibration curve (y = 0.0016x, r^2^ = 0.9916).

#### 2.6.2. Total Carbohydrate Content (TChC)

TChC was determined using the phenol-sulfuric acid method described by [31], with modifications. Aliquots (50 mg of powdered leaves) were extracted with 20 mL of distilled water. After centrifugation, 2 mL of the supernatant was mixed with 50 μL of 80% phenol and 5 mL of concentrated sulfuric acid. After 10 min, the mixture was gently agitated and incubated in a water bath at 25–30 °C for 15 min. After cooling, samples were transferred to microplates and analyzed in triplicate. Absorbance was measured at 490 nm using a microplate reader (ThermoPlate, model P-reader; Dallas, TX, USA). Galactose was used as the standard at concentrations ranging from 7.80 to 500 μg mL^−1^ and TChC was calculated from the calibration curve (y = 0.0025x, r^2^ = 0.9992).

Because total carbohydrate content (TChC), total soluble sugars (TSS), and total starch (TS) were determined using independent extraction procedures and analytical methods, their values represent distinct carbohydrate fractions and should not be interpreted as additive components of a single carbohydrate pool.

### 2.7. Determination of Nitrogen-Related Metabolites

#### 2.7.1. Soluble Protein Content (SPC)

SPC was quantified using the Bradford assay [32], with modifications. Proteins were extracted from 200 mg of powdered leaves with 10 mL of phosphate-buffered saline (PBS; pH 7.0). Samples were homogenized, incubated for 15 min, and centrifuged. An aliquot of 100 μL of the supernatant was mixed with 5 mL of Bradford solution (1 mL of Bradford reagent in 4 mL of water). After 5 min, samples were transferred to microplates and analyzed in triplicate. Absorbance was measured at 595 nm using a microplate reader (ThermoPlate, model P-reader; Dallas, TX, USA). Bovine serum albumin was used as the standard at concentrations ranging from 3.90 to 62.5 μg mL^−1^ and SPC was calculated from the calibration curve (y = 0.0032x, r^2^ = 0.9772).

#### 2.7.2. Total Amino Acid Content (TAAC)

TAAC was determined according to [33], with modifications. Amino acids were extracted from 100 mg of powdered leaves with 2 mL of distilled water. After centrifugation, 1 mL of the supernatant was mixed with 3 mL of 2% ninhydrin solution in PBS (pH 7.0). The reaction mixture was heated in a water bath at 100 °C for 30 min and then cooled. Aliquots were transferred to microplates and analyzed in triplicate. Absorbance was measured at 570 nm using a microplate reader (ThermoPlate, model P-reader; Dallas, TX, USA). Proline was used as the standard at concentrations ranging from 1.95 to 499.20 μg mL^−1^ and TAAC was calculated from the calibration curve (y = 0.0026x, r^2^ = 0.9962).

### 2.8. Grain Yield Evaluation

Grains were harvested when approximately 90% of the panicles had reached physiological maturity and grain moisture was about 22%. After harvest, grains were cleaned and dried to 13% moisture. Productive performance was assessed at the plant and field scales using thousand-grain weight (TGW) and grain yield per area, according to [34]. Yield components were evaluated by randomly collecting 20 panicles from each replicate (*n* = 80 panicles per treatment) and determining the number of filled and empty grains per panicle. Grain yield per area was calculated by extrapolating the grain mass from the harvested plot area to a hectare basis and expressed as kg ha^−1^.

### 2.9. Grain Processing and Quality Analyses

Post-harvest evaluations, including grain processing yield and grain quality, were performed according to the protocol established by [34]. Briefly, a 105 g sample of rough rice from each experimental unit was processed using a laboratory rice mill (PAZ-2/DTA model; Zaccaria, São Paulo, Brazil) for 1 min to remove the husk, followed by 30 s of polishing. Whole polished grains and broken grains were subsequently separated with a no. 1 trieur, weighed, and expressed as percentages of head rice yield and broken grains, respectively. Grain appearance traits, including the incidence of chalky grains and white belly, were also evaluated and expressed as percentages.

Grain nutritional composition, including protein, lipid, fiber, ash, and starch contents, was determined using Near-Infrared Spectroscopy (NIRS) [35]. NIR spectra were obtained using a FOSS NIRS DS2500 L analyzer (FOSS Analytics, Hillerød, Denmark), and grain composition was estimated using previously validated calibration models for rice grain constituents.

### 2.10. Statistical Analysis

Data were first tested for normality using InfoStat version 2020 (InfoStat, Córdoba, Argentina). When the normality assumption was met, data were subjected to analysis of variance (ANOVA), and means were compared using Tukey’s test at *p* < 0.05. When the data did not meet this assumption, pairwise comparisons were performed using Kruskal–Wallis test at *p* < 0.05. Mean temperatures inside and outside the greenhouse structures during the 16-day HS period were compared using Student’s *t* test. All analyses were performed in SPSS (version 23.0; IBM Corp., Armonk, NY, USA). Results are means ± standard error (SE), and different letters indicate significant differences among treatments.

## 3. Results and Discussion

### 3.1. Environmental Conditions and Heat Stress Imposition

**Figure 3:**
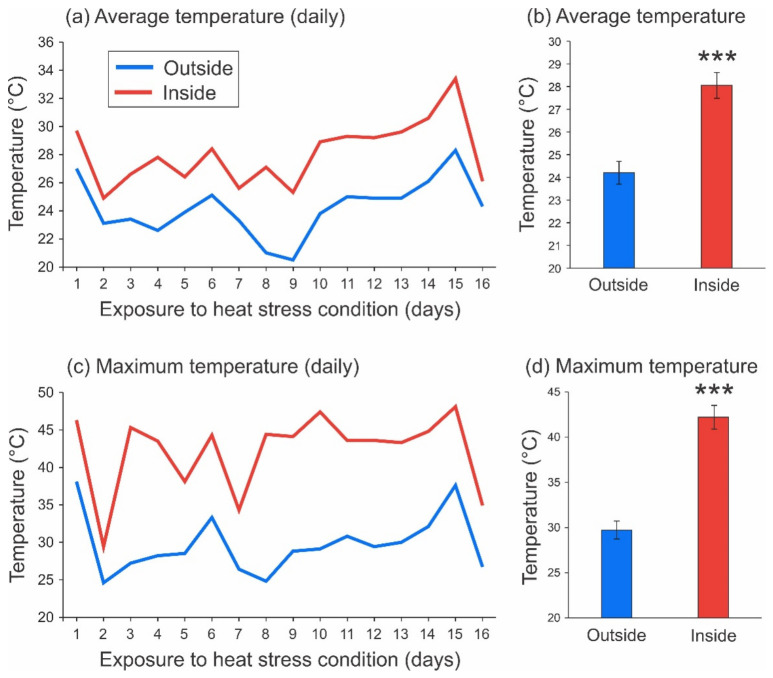
Air temperature during the heat stress experiment. (**a**) Daily mean air temperature recorded inside (red line) and outside (blue line) the temporary greenhouse structures during the 16-day heat-stress period imposed at the reproductive stage (R_4_). (**b**) Mean air temperature over the stress period, showing significantly higher temperatures inside the greenhouse structures compared to ambient conditions. (**c**) Daily maximum air temperature recorded inside (red line) and outside (blue line) the greenhouse structures during the stress period. (**d**) Mean maximum temperature over the stress period, showing higher peak temperatures inside the greenhouse structures. Data in (**b**,**d**) are means ± standard error (SE). Asterisks (***) indicate significant differences between inside and outside conditions (*p* < 0.001).

The environmental conditions followed typical seasonal patterns, with daily average temperatures ranging from 20 to 27 °C, and daily maximum temperatures reaching 24 to 38 °C (Figure 3a,c). These conditions are within the optimal to moderately permissive range for rice normal development, particularly during the reproductive stage. The temporary greenhouses effectively imposed HS during the reproductive stage of rice. During the 16-day exposure period, the average air temperature inside the structures was 3.9 °C higher than under ambient field conditions, reaching 28.1 °C compared with 24.2 °C outside (Figure 3a,b). HS also sharply increased daily maximum temperature. Inside the temporary greenhouses, maximum temperatures frequently exceeded 40 °C and reached peaks above 45 °C, whereas outside values averaged 29.7 °C (Figure 3d). Overall, the maximum temperature under HS was 12.5 °C higher than under non-stress conditions.

The temporal dynamics of temperature also indicate that HS was not only more intense but consistently sustained over the experimental period. While outside temperatures showed moderate fluctuations typical of field conditions, the greenhouse structures amplified both baseline and peak temperatures, increasing thermal load during both daytime and daily peaks. From a physiological perspective, temperatures above 35 °C during the reproductive stage are known to impair pollen viability, fertilization, and spikelet fertility in rice [12,14]. Thus, while the first experiment under non-stress conditions represents a baseline without thermal constraint, the thermal regime imposed in the second experiment can be classified as a severe HS. This contrast between ambient and HS conditions provided a suitable framework to assess the context-dependent effects of S-CDs on rice reproductive performance.

### 3.2. Antioxidant Metabolism in Response to S-CD Application

Under the non-stress field condition, S-CD application increased antioxidant-related secondary metabolites at the R_4_ stage. Total phenolic content (TPhnlcC) and total flavonoid content (TFC) increased by approximately 15% and 48%, respectively, compared with the control (Table 1). Phenolic compounds and flavonoids are central components of the plant non-enzymatic antioxidant system, contributing to redox homeostasis, cellular signaling, and protection against oxidative damage through reactive oxygen species (ROS) scavenging [36]. Recent studies further demonstrate that reactive oxygen and nitrogen species (ROS/RNS) act not only as toxic byproducts of stress metabolism, but also as integrated signaling molecules that regulate stress perception, acclimation, metabolic reprogramming, and reproductive responses under adverse environmental conditions [37,38]. Thus, modulation of antioxidant-related metabolism by S-CDs may also reflect adjustments in redox-mediated signaling networks during stress acclimation. Their accumulation is often associated with metabolic adjustments during developmental transitions, response to environmental constraints, and periods of high metabolic demand [36,39]. In rice, the late reproductive stage involves intense source-sink reorganization and enhanced antioxidant capacity may contribute to maintaining metabolic stability and protecting photosynthetic and reproductive tissues [40,41].

Similar increases in phenolic metabolism and antioxidant responses have been reported in plants exposed to carbon-based nanomaterials under controlled conditions, including studies with carbon nanotubes, graphene, and CDs [42,43]. These responses were linked to redox modulation, activation of stress-related pathways, and priming-like effects. As total antioxidant capacity measured by the DPPH assay and total carotenoid content (TCrtnC) did not differ from the control (Table 1), the response appears to reflect a selective shift in secondary metabolism rather than a broad increase in antioxidant activity.

Under HS condition, S-CD application did not affect any of the redox-related variables evaluated (Table 1). This may indicate that thermal stress had already constrained antioxidant metabolism at this stage, limiting further modulation by S-CD application. Our results contrast with reports in lettuce (*Lactuca sativa* L.) and Chinese cabbage (*Brassica rapa* L.), in which CDs improved thermotolerance through modulation of antioxidant systems and stress-responsive pathways [37,44,45].

Together, these results indicate that the effects of S-CD application on antioxidant metabolism depend on the environmental context, with selective accumulation of phenolic compounds and flavonoid under non-stress conditions and no clear response under HS. However, we cannot exclude the possibility that the effects of S-CDs depend on HS intensity and the physiological state of the plant.

### 3.3. Effects of S-CDs on Primary Carbon Metabolism

Under non-stress field conditions, S-CD application influenced primary carbon metabolism, increasing total carbohydrate content (TChC), total soluble sugars (TSS), and total starch (TS) by 34%, 9%, and 43%, respectively, compared with the control (Table 2). These results suggest a positive effect of S-CDs on carbon assimilation and partitioning during the reproductive stage under favorable conditions. Similar responses have been reported in plants treated with carbon-based nanomaterials. In maize (*Zea mays* L.), foliar application of nitrogen-doped carbon dots (N-CDs) increased net photosynthetic rate, carbohydrate content, biomass, grain yield, and thousand-grain weight during the reproductive stage, possibly through the upregulation of sucrose transporter genes [46]. In rice, CDs have also been linked to increased CO_2_ assimilation and improved growth, suggesting enhanced photosynthetic efficiency and carbon use [47]. Additional evidence indicates that N-CDs can stimulate starch metabolism and sucrose translocation, thereby improving photoassimilate partitioning and yield under nutrient-limited conditions [48,49].

Under the HS condition, S-CD-treated plants showed 42% lower TChC than plants subjected to HS alone, while TSS and TS did not differ between treatments (Table 2). This result suggests that S-CD application altered overall carbon balance under thermal stress, but without detectable changes in the major carbohydrate fractions evaluated. This pattern is consistent with preferential use of assimilated carbon for metabolic maintenance and stress acclimation rather than for accumulation as reserve compounds [50]. In rice, HS can disrupt carbon metabolism by impairing photosynthesis, increasing respiratory demand, and altering source-sink relationships, particularly during the reproductive stage [51,52].

### 3.4. Effects of S-CD on Nitrogen-Related Metabolites

Under the non-stress field condition, S-CD application did not affect soluble protein content (SPC) or total amino acid content (TAAC) (Table 3). Under the HS condition, TAAC also remained unchanged, whereas SPC decreased by 21% in S-CD-treated plants compared to the HS alone (Table 3). These results suggest that S-CD application did not increase nitrogen accumulation, but may have altered nitrogen partitioning under thermal stress. The reduction in protein content without changes in total amino acids points to adjustments in protein turnover or allocation rather than changes in nitrogen assimilation. This response is consistent with stress-induced reorganization of nitrogen metabolism, in which protein degradation and amino acid remobilization help maintain metabolic homeostasis under adverse conditions [11].

The nitrogen-related responses were consistent with the patterns observed for carbon metabolism, suggesting coordinated metabolic adjustments under HS. Although we did not assess the underlying mechanisms, the lack of major changes in amino acid pools suggests that the effects of S-CD application appear to be more closely related to protein dynamics rather than to bulk nitrogen accumulation. Similar responses have been reported for carbon-based nanomaterials under abiotic stress conditions, where their effects are associated more with metabolic and redox regulation than with direct increases in nutrient accumulation [53,54]. Together, these results support a context-dependent effect of S-CD application on plant metabolism under HS conditions.

### 3.5. Productive Performance and Grain Quality

Under the non-stress field condition, S-CD application did not affect productive performance, including thousand-grain weight (TGW), spikelet sterility, and grain yield (Table 4). These results indicate that, under favorable conditions, S-CD application did not improve productivity. In contrast, grain physical quality was affected. S-CD-treated plants showed higher head rice yield (14%) and a greater proportion of whole kernels (12%), but also a marked increase in the proportion of chalky grains (283%) (Table 4). No significant differences were observed for broken grains or white-belly grains. These results collectively suggest that S-CDs affected some traits related to grain processing quality under ambient conditions, but without consistent improvements in overall grain quality.

Under the HS condition, S-CD-treated plants showed a significant increase in whole kernels (9%) and broken grains (177%), along with lower spikelet sterility (29%) and higher grain yield (34%) compared to plants exposed to HS alone (Table 4). TGW did not differ among treatments. These results suggest that S-CD application partly mitigated the negative effects of HS on reproductive performance, contributing to higher yield under adverse conditions. HS during anthesis severely affects pollen viability, anther dehiscence, fertilization, and assimilate allocation to developing spikelets, and the reduced spikelet sterility observed in S-CD-treated plants suggests a possible protective effect on these reproductive processes. However, the simultaneous increase in broken grains indicates that this improvement in yield was not accompanied by better grain physical integrity.

Under non-stress conditions, S-CD application altered grain nutritional composition, increasing protein (21%), lipid (381%), and starch (25%) contents while reducing crude fiber by 62% and ash (69%) (Table 5). Under HS condition, S-CD-treated plants also showed higher protein (26%), lipid (254%), and starch (23%) contents and lower crude fiber (65%) and ash (67%) than plants subjected to HS alone (Table 5). These changes suggest that S-CD application modified grain biochemical composition under both conditions, although the response was more relevant under HS because it was accompanied by higher grain yield.

Previous studies have shown that carbon-based nanomaterials can enhance grain filling and productivity through improved photosynthesis, assimilate translocation, and the upregulation of sucrose transport and metabolism-related genes [47,48]. Similar responses have been reported in maize, soybean, and tomato, particularly under stress conditions [55,56]. In our study, S-CD application did not improve productivity under non-stress conditions, but under HS it reduced spikelet sterility and increased grain yield. However, these benefits were accompanied by trade-offs in grain physical quality, reinforcing the context-dependent nature of S-CD effects on rice performance. These results reinforce that metabolic stimulation alone does not necessarily translate into higher productivity, particularly under favorable conditions in which source-sink limitations and reproductive constraints may already be minimized. Thus, the metabolic responses induced by S-CDs under non-stress conditions may reflect physiological acclimation or buffering rather than effective conversion into agronomic advantages. Recent advances in single-cell and spatial transcriptomics further reinforce this complexity by demonstrating that stress responses can vary substantially among tissues and cell types, generating heterogeneous regulatory dynamics that may remain undetected in bulk metabolic analyses [57]. These high-resolution approaches may therefore help explain why metabolic modulation does not consistently translate into predictable agronomic outcomes under field conditions.

**Figure 4:**
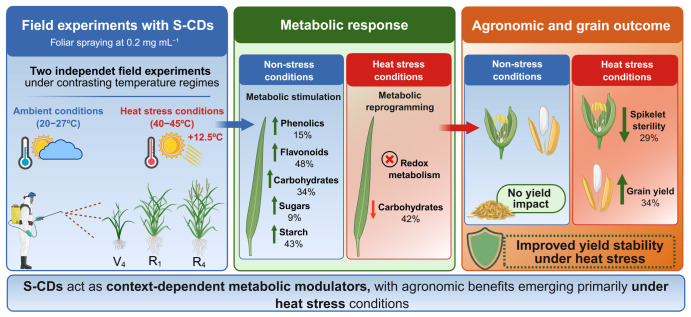
Context-dependent effects of Spirulina-derived carbon dots (S-CDs) on rice metabolism and agronomic performance under non-stress and heat stress field conditions.

To integrate the metabolic and agronomic responses observed under contrasting environmental conditions, a schematic model summarizing the effects of S-CDs is presented in Figure 4. Under non-stress conditions, S-CD application promoted metabolic stimulation, characterized by increased accumulation of phenolic compounds, flavonoids, and carbohydrates, without consistent effects on grain yield. In contrast, under HS, S-CDs induced a distinct metabolic reprogramming, associated with reduced spikelet sterility and increased grain yield. These results indicate that the agronomic benefits of S-CDs emerge primarily under stress conditions, supporting their role as context-dependent metabolic modulators that enhance yield stability.

## 4. Conclusions

This study advances our understanding of how S-CDs affect rice under field conditions. Overall, S-CD application induced context-dependent responses, with limited agronomic relevance under favorable conditions. Although S-CDs altered metabolic traits under favorable conditions, these changes did not improve grain yield or its components. Under HS during the reproductive stage, however, S-CD application reduced spikelet sterility and increased grain yield, indicating potential to support reproductive performance under adverse thermal conditions. Although S-CD application reduced spikelet sterility and increased grain yield under HS, the molecular and signaling pathways underlying this response remain to be elucidated. Future studies integrating transcriptomics, proteomics, metabolomics, reproductive biology, and time-resolved physiological analyses should investigate whether S-CDs modulate ROS signaling, antioxidant enzyme activity, heat-shock protein accumulation, hormone-mediated stress responses, pollen viability, anther dehiscence, source-sink regulation, and assimilate partitioning during rice reproductive development under high temperature. The integrative framework presented in Figure 4 demonstrates that metabolic responses alone are insufficient to predict agronomic outcomes, underscoring the central role of environmental context in shaping the effectiveness of nanobiostimulants. These findings suggest that S-CDs act less as general growth promoters and more as context-dependent modulators of plant responses to environmental stress, which is relevant under increasingly frequent and intense climate-related stress conditions.

## Figures and Tables

**Table 1:** Effects of foliar application of Spirulina-derived carbon dots (S-CDs) or the commercial product Arbolina (0.2 mg mL^−1^) on total phenolic content (TPhnlcC), total flavonoid content (TFC), total carotenoid content (TCrtnN), and antioxidant capacity determined by the DPPH assay in fully expanded rice leaves collected from the position immediately below the flag leaf at the reproductive stage (R4, anthesis) under non-stress conditions, or after 7 d of heat stress (HS) exposure. Data are means ± SE of four biological replicates. Significant differences (Tukey’s test for parametric data or Kruskal–Wallis for non-parametric data, *p* < 0.05) among treatments are indicated by different letters in each column. DW: dry weight.

Conditions	Treatments	TPhnlcC (mg g^−1^ DW)	TFC (mg g^−1^ DW)	TCrtnN (mg g^−1^ DW)	DPPH (%)
**Non-stress**	**Control**	6.64 ± 0.12 b	3.97 ± 0.11 b	7.34 ± 0.53 a	71.62 ± 0.59 a
	**S-CDs**	7.65 ± 0.12 a	5.86 ± 0.18 a	6.40 ± 0.35 a	73.03 ± 0.56 a
	**Arbolina**	6.84 ± 0.15 b	5.33 ± 0.26 a	6.84 ± 0.12 a	72.98 ± 0.67 a
**Heat stress (HS)**	**HS**	4.65 ± 0.22 b	5.01 ± 0.29 a	3.28 ± 0.44 a	74.69 ± 1.11 a
	**HS + S-CDs**	5.39 ± 0.24 ab	4.53 ± 0.35 a	3.87 ± 0.37 a	72.64 ± 0.87 a
	**HS + Arbolina**	5.67 ± 0.20 a	5.79 ± 0.25 a	3.30 ± 0.17 a	74.96 ± 0.64 a

**Table 2:** Effects of foliar application of Spirulina-derived carbon dots (S-CDs) or the commercial product Arbolina (0.2 mg mL^−1^) on total carbohydrate content (TChC), total soluble sugars (TSS), and total starch (TS) in fully expanded rice leaves collected from the position immediately below the flag leaf at the reproductive stage (R4, anthesis) under non-stress conditions, or after 7 d of heat stress (HS) exposure. Data are means ± SE of four biological replicates. Significant differences (Tukey’s test for parametric data or Kruskal–Wallis for non-parametric data, *p* < 0.05) among treatments are indicated by different letters in each column. DW: dry weight.

Conditions	Treatments	TChC (mg g^−1^ DW)	TSS (mg g^−1^ DW)	TS (mg g^−1^ DW)
**Non-stress**	**Control**	58.86 ± 2.23 b	270.20 ± 5.84 b	34.65 ± 1.81 b
	**S-CDs**	78.68 ± 0.76 a	295.48 ± 4.49 a	49.52 ± 1.16 a
	**Arbolina**	63.52 ± 2.75 b	289.73 ± 4.36 a	44.88 ± 2.90 a
**Heat stress (HS)**	**HS**	154.88 ± 3.88 a	295.12 ± 4.44 b	14.51 ± 0.65 b
	**HS + S-CDs**	89.69 ± 1.86 c	292.67 ± 3.88 b	13.77 ± 0.56 b
	**HS + Arbolina**	118.90 ± 2.90 b	310.87 ± 2.63 a	28.01 ± 2.37 a

**Table 3:** Effects of foliar application of Spirulina-derived carbon dots (S-CDs) or the commercial product Arbolina (0.2 mg mL^−1^) on soluble protein content (SPC) and total amino acid content (TAAC) in fully expanded rice leaves collected from the position immediately below the flag leaf at the reproductive stage (R4, anthesis) under non-stress conditions, or after 7 d of heat stress (HS) exposure. Data are means ± SE of four biological replicates. Significant differences (Tukey’s test for parametric data or Kruskal–Wallis for non-parametric data, *p* < 0.05) among treatments are indicated by different letters in each column. DW: dry weight.

Conditions	Treatments	SPC (mg g^−1^ DW)	TAAC (mg g^−1^ DW)
**Non-stress**	**Control**	0.98 ± 0.09 a	16.06 ± 0.46 a
	**S-CDs**	1.04 ± 0.07 a	18.20 ± 0.51 a
	**Arbolina**	1.03 ± 0.10 a	18.04 ± 1.47 a
**Heat stress (HS)**	**HS**	2.16 ± 0.11 a	28.33 ± 1.07 a
	**HS + S-CDs**	1.71 ± 0.14 b	24.66 ± 0.48 a
	**HS + Arbolina**	0.90 ± 0.11 c	25.19 ± 1.11 a

**Table 4:** Effects of foliar application of Spirulina-derived carbon dots (S-CDs) or the commercial product Arbolina (0.2 mg mL^−1^) on productivity-related parameters determined at harvest in rice plants grown under non-stress or heat stress (HS) conditions. Evaluated traits included whole kernels (%), head rice yield (%), broken grains (%), chalky grains (%), white-belly grains (%), thousand-grain weight (g), spikelet sterility (%), and grain yield (kg ha^−1^). Data are means ± SE of four biological replicates. Significant differences (Tukey’s test for parametric data or Kruskal–Wallis for non-parametric data, *p* < 0.05) among treatments are indicated by different letters in each column.

Conditions	Treatments	Whole Kernels (%)	Head Rice Yield (%)	Broken Grains (%)	Chalky Grains (%)	White-Belly Grains (%)	Thousand-Grain Weight (g)	Spikelet Sterility (%)	Grain Yield (kg ha^−1^)
**Non-stress**	**Control**	70.71 ± 0.77 b	66.25 ± 0.50 b	3.86 ± 0.32 a	0.41 ± 0.04 b	19.55 ± 3.18 a	26.39 ± 0.16 a	18.18 ± 0.31 b	11,047.16 ± 79.95 a
	**S-CDs**	78.94 ± 0.24 a	75.47 ± 0.35 a	3.72 ± 0.20 a	1.57 ± 0.19 a	11.04 ± 0.71 ab	26.26 ± 0.07 a	16.00 ± 0.58 b	11,528.64 ± 268.19 a
	**Arbolina**	78.93 ± 0.17 a	75.27 ± 0.43 a	3.93 ± 0.35 a	0.49 ± 0.08 ab	8.00 ± 0.70 b	26.12 ± 0.11 a	24.33 ± 1.67 a	11,516.00 ± 359.93 a
**Heat stress (HS)**	**HS**	71.95 ± 0.10 b	64.17 ± 1.91 a	2.26 ± 0.22 b	1.65 ± 0.33 a	28.59 ± 7.90 a	25.48 ± 0.07 a	32.67 ± 1.45 a	8239.90 ± 803.85 b
	**HS + S-CDs**	78.55 ± 0.20 a	61.63 ± 1.24 a	6.28 ± 0.63 a	1.35 ± 0.50 a	15.31 ± 0.96 ab	25.92 ± 0.12 a	23.00 ± 2.65 b	11,085.09 ± 220.35 a
	**HS + Arbolina**	78.85 ± 0.38 a	63.00 ± 0.09 a	4.94 ± 0.36 a	0.94 ± 0.15 a	12.44 ± 0,48 b	25.65 ± 0.15 a	26.33 ± 2.33 ab	11,064.87 ± 408.85 a

**Table 5:** Effects of foliar application of Spirulina-derived carbon dots (S-CDs) or the commercial product Arbolina (0.2 mg mL^−1^) on grain quality-related parameters determined at harvest in rice plants grown under non-stress and heat stress (HS) conditions. Evaluated traits included percentages of proteins, lipids, crude fibers, ash, and starch. Data are means ± SE of four biological replicates. Significant differences (Tukey’s test for parametric data or Kruskal–Wallis for non-parametric data, *p* < 0.05) among treatments are indicated by different letters in each column.

Conditions	Treatments	Proteins (%)	Lipids (%)	Crude Fibers (%)	Ash (%)	Starch (%)
**Non-stress**	**Control**	5.33 ± 0.05 b	0.43 ± 0.06 b	5.58 ± 0.01 a	3.28 ± 0.03 a	58.78 ± 0.13 b
	**S-CDs**	6.48 ± 0.07 a	2.07 ± 0.04 a	2.10 ± 0.01 b	1.03 ± 0.01 b	73.68 ± 0.16 a
	**Arbolina**	6.52 ± 0.14 a	2.04 ± 0.03 a	2.10 ± 0.01 b	0.98 ± 0.01 b	73.81 ± 0.17 a
**Heat stress (HS)**	**HS**	5.96 ± 0.28 b	0.59 ± 0.09 b	5.93 ± 0.05 a	3.26 ± 0.07 a	59.54 ± 0.09 b
	**HS + S-CDs**	7.51 ± 0.28 a	2.09 ± 0.05 a	2.09 ± 0.01 b	1.07 ± 0.03 b	73.08 ± 0.33 a
	**HS + Arbolina**	7.33 ± 0.17 a	2.12 ± 0.03 a	2.09 ± 0.01 b	1.01 ± 0.01 b	73.28 ± 0.19 a

## Data Availability

The data that support the findings of this study are available from the corresponding author upon reasonable request.

## Supplementary Materials

The following supporting information can be downloaded at https://www.mdpi.com/article/10.3390/plants15111657/s1, Table S1. Chemical properties of the soil (0–20 cm depth) prior to experiment establishment.
