# Supplementary material for: Spirulina-Derived Carbon Dots Promote Context-Dependent Effects on Rice Metabolism, Yield, and Grain Quality Under Non-Stress and Heat Stress Conditions
Source: Plants (Basel). 2026 May 28;15(11):1657. doi: 10.3390/plants15111657 (PMC13259005; doi:10.3390/plants15111657)
Supplement: Supplementary file 1 [file plants-15-01657-s001.zip › plants-4303431-supplementary.pdf]

Supplementary Table 1. Chemical properties of the soil (0–20 cm depth) prior to experiment establishment.

Soil property	Value	Unit
pH (H_2O, 1:1)	5.1	–
Organic matter (OM)	1.2	%
Clay content	14	%
Available P (Mehlich-1)	14.6	mg dm^{-3}
Exchangeable K	60	mg dm^{-3}
Sulfur (S)	10.6	mg dm^{-3}
Exchangeable Ca	1.5	cmolc dm^{-3}
Exchangeable Mg	0.4	cmolc dm^{-3}
Exchangeable Al	0.4	cmolc dm^{-3}
Potential acidity (H + Al)	2.8	cmolc dm^{-3}
Effective cation exchange capacity (ECEC)	2.4	cmolc dm^{-3}
